# A fine-scale dissection of the DNA double-strand break repair machinery and its implications for breast cancer therapy

**DOI:** 10.1093/nar/gku284

**Published:** 2014-05-12

**Authors:** Chao Liu, Sriganesh Srihari, Kim-Anh Lê Cao, Georgia Chenevix-Trench, Peter T. Simpson, Mark A. Ragan, Kum Kum Khanna

**Affiliations:** 1 Institute for Molecular Bioscience, The University of Queensland, St. Lucia QLD 4072, Australia; 2 Queensland Facility for Advanced Bioinformatics, The University of Queensland, St. Lucia 4072, Australia; 3 QIMR-Berghofer Medical Research Institute, Herston, Brisbane, QLD 4029, Australia; 4 The University of Queensland Centre for Clinical Research, Herston, Brisbane, QLD 4029, Australia

## Abstract

DNA-damage response machinery is crucial to maintain the genomic integrity of cells, by enabling effective repair of even highly lethal lesions such as DNA double-strand breaks (DSBs). Defects in specific genes acquired through mutations, copy-number alterations or epigenetic changes can alter the balance of these pathways, triggering cancerous potential in cells. Selective killing of cancer cells by sensitizing them to further DNA damage, especially by induction of DSBs, therefore requires careful modulation of DSB-repair pathways.

Here, we review the latest knowledge on the two DSB-repair pathways, homologous recombination and non-homologous end joining in human, describing in detail the functions of their components and the key mechanisms contributing to the repair. Such an in-depth characterization of these pathways enables a more mechanistic understanding of how cells respond to therapies, and suggests molecules and processes that can be explored as potential therapeutic targets. One such avenue that has shown immense promise is *via* the exploitation of synthetic lethal relationships, for which the *BRCA1–PARP1* relationship is particularly notable. Here, we describe how this relationship functions and the manner in which cancer cells acquire therapy resistance by restoring their DSB repair potential.

## INTRODUCTION

Most deoxyribonucleic acid (DNA)-damaging chemotherapeutic agents directly or indirectly cause DNA double-strand breaks (DSBs), which are highly lethal lesions sufficient to kill cells by inactivating essential genes or, in metazoans, by triggering apoptosis (1,2). The key to highly selective cancer therapies therefore lies in exploiting the distinctive molecular and cellular traits that sensitize only cancer cells to these agents.

Cancer is a disease of genomic instability and cancer cells differ genetically from normal cells in their ability to repair their DNA. Consequently, if these differences can be exploited to induce a high level of DNA damage, which can nonetheless be repaired in normal cells, then cancer cells can be selectively forced into DNA-damage-induced apoptosis. DNA-damage response (DDR) pathways offer molecular targets to exploit cancer-specific traits and through their precise modulation, cancer cells can be selectively sensitized to DSB-inducing drugs.

Cells have evolved an intricate assembly of interlocking mechanisms that repair DSBs efficiently or, if the damage cannot be repaired, commit the cells to apoptosis. Extensive studies mapping mutational landscapes of cancers have linked specific defects in DSB-repair pathways to ‘driver’ events in breast and other cancers (3,4). It is also now established that cancer cells become drug-resistant and retain their proliferative potential by modulating their DSB-repair potential (5). Therefore in-depth characterization of DSB-repair pathways and deciphering their connection to tumorigenic activity is critical to understand the basis of cancer and develop effective therapies.

In the following section, we describe the basic mechanisms underlying DSB-repair and associated sub-pathways, from sensing of DNA damage and recruitment of early-response factors through to repair and the re-joining of DNA ends. In the subsequent section, by associating specific genes and mechanisms in these pathways to cancerous potential particularly for breast cancer, we outline how this information can be harnessed to improve cancer therapy, focusing on a promising strategy called *synthetic lethality*.

## DNA-damage response (DDR)

The detection of DSBs activates a sequence of closely linked cellular events, designated the DDR, consisting of cell-cycle checkpoint activation, chromatin modification, transcriptional changes, DNA repair, or apoptotic cell death in cases where the damage cannot be repaired [see (1,6–8) for more details]. The principal function of this regulatory network is to maximize the likelihood that any genetic lesion incurred is faithfully repaired prior to being transmitted to progeny during DNA replication or mitotic cell division. Critical regulators of cell cycle checkpoints include the ATM (ataxia telangiectasia mutated) and ATR (ataxia telangiectasia and Rad3 related) protein kinases, which act in concert or independently to deal with DNA damage in the cell (9,10). A large-scale proteomics screen identified >700 proteins phosphorylated by ATM and/or ATR in response to genotoxic stress, demonstrating the broad impact of DNA damage on cellular signalling (11–13). The checkpoint functions of ATR and ATM are mediated, in part, by a pair of checkpoint effector kinases termed CHK1 (checkpoint kinase 1) and CHK2 (checkpoint kinase 2) [reviewed in (14)]. Another direct target of ATM phosphorylation relevant to G1 phase cell cycle arrest is p53 (9), one of the most important tumour suppressors. Together with its key target p21, p53 plays an important role in inducing cell cycle arrest and regulating the balance between repair and survival of the cell or apoptosis [(15,16); recently reviewed in (8)]. In addition to the classical transducers (ATM and ATR) and effector kinases (CHK1 and CHK2), stress-activated p38 SAPK (stress-activated protein kinase) and its downstream target MAPKAP-kinase 2 (mitogen-activated protein kinase-activated protein kinase 2) (17,18) and tyrosine kinases such as Abl (Abelson murine leukemia) play an important role in coordinating the DDR of higher eukaryotic cells (19,20). Description of all the DDR pathways is beyond the scope of this article, and the reader is referred to several excellent reviews (1,6,7,21); only salient features of DSB repair will be highlighted here.

## DSB REPAIR

Homologous recombination (HR) and Non-homologous end joining (NHEJ) are the two main DSB repair pathways. HR restores the original DNA sequence at DSB sites using a template sequence from a sister chromatid or a homologous chromosome to direct the error-free repair of DSBs, and is restricted to the S and G2 phases of the cell cycle. In addition to DSB repair, HR is also involved in the resolution of stalled replication forks and in the generation of genetic diversity through mitotic and meiotic recombination (22,23). By contrast, NHEJ directly joins the two ends of a DSB, regardless of the sequence template at the exposed ends of the break, making it error-prone but available at all times during the cell cycle. NHEJ is also involved in the maturation of immune cells through V(D)J recombination and class-switch recombination (CSR) (24). The major steps in DSB-mediated repair pathways are discussed here.

### DNA damage-induced chromatin relaxation

In most eukaryotic cells the DNA is tightly packaged into the DNA-protein complex known as chromatin, which presents a significant barrier for DSB-repair proteins to access and repair DNA breaks. The dynamic restructuring of chromatin surrounding the lesion including modifications of histone tails and remodelling of chromatin by remodelling factors allow HR and NHEJ machinery access to the damaged DNA (25–27). The most prominent chromatin modification after DSB induction is phosphorylation of H2AX (a histone H2A variant), which plays a primary role in the DNA damage repair by facilitating the access of HR factors to sites of DNA damage (next section).

In response to a DSB, the chromatin surrounding the DSB is rapidly PARylated (modified by covalent addition of poly-ADP ribose, or PAR), a reaction catalysed by PARP1 [poly (ADP-ribose) polymerase 1] (28). This creates PAR chains at DSBs, allowing rapid and transient accumulation of the NuRD, PcG (polycomb group) and ALC1 remodelling complexes through interaction with PAR (28–30), and of the KAP-1/HP1 complex possibly through interaction with PAR at break sites [reviewed in (25)]. The NuRD complex is required for subsequent steps in DDR such as efficient marking of DNA-damage site with ubiquitin by RNF8 (ring finger protein 8) and RNF168 (ring finger protein 168), and also for recruitment of BRCA1 to damaged DNA (31). PcG proteins exist as two main complexes, PRC1 and PRC2 (polycomb repressive complexes 1 and 2), which are recruited to DSB sites in a PARP-dependent manner. PRC1 can monoubiquitinate histone H2A at sites of DSBs, and PRC1-mediated monoubiquitination is required for subsequent RNF8- and/or RNF168-mediated polyubiquitination at DSBs (32–34). ALC1 may have a role in repositioning DSB-flanking nucleosomes, and in stabilizing the chromatin structure for further DSB processing and repair, while the KAP-1/HP1 complex may promote the unpacking of heterochromatin, thereby facilitating repair of heterochromatic DSBs (26). These complexes are retained at DSBs for only a short period of time, and then rapidly released from the chromatin, potentially through dePARylation by PARG (polyADP-ribose glycohydrolases) (25). The requirement of PARG for efficient DNA repair suggests that the presence of PAR at sites of DNA of damage must be tightly regulated.

Subsequent DSB signalling, including ATM activation and phosphorylation of histone H2AX, recruits MDC1 (mediator of DNA damage checkpoint 1) which then interacts with and loads another chromatin-remodelling complex, NuA4, onto chromatin adjacent to DSBs (35). Loading of NuA4 catalyses the exchange of H2A for H2A.Z through the p400 component of NuA4 [(36); reviewed in (25)]. This reaction is required for the acetylation of histone H4 by the TIP60 (also known as KAT5) component of NuA4, leading to the relaxation of DNA in proximity to DSBs [reviewed in (25,37)].

Chromatin relaxation in both HR and NHEJ also involves ubiquitination of histone H2B by the heterodimer consisting of RNF20 (ring finger protein 20) and RNF40 (ring finger protein 40) in an ATM-dependent manner (38). Two tumour suppressors, CDC73 (cell division cycle 73) (39) and Smurf2 (smad ubiquitin regulatory factor 2) (40), have been reported to regulate this ubiquitination reaction, and this may represent a major mechanism by which mutations in these tumour suppressors exert their tumorigenic effect (39,40).

### Homologous Recombination (HR)

HR occurs through a series of steps involving DSB-induced chromatin relaxation; recruitment of early HR factors to site of DSBs; DSB end resection; formation of the D loop; processing of the D loop or Holliday junctions; and the single-strand annealing (SSA) sub-pathway. We consider these in order.

#### Recruitment of early HR factors to DSBs

**Figure 1.**
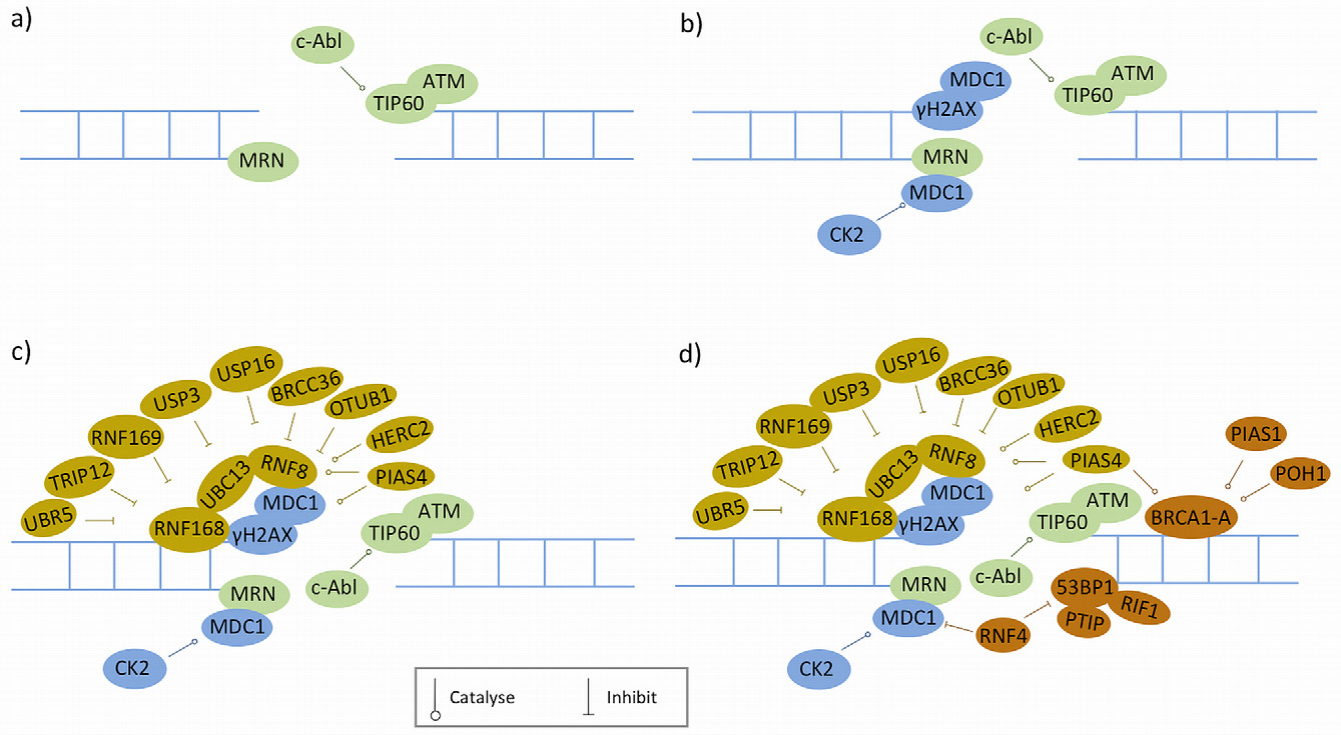
Recruitment of early homologous recombination (HR) factors to double-strand breaks (DSBs). Proteins represented in different colours are recruited at different times: **a**) The MRN (MRE11–RAD50–NBS1) complex recognizes and binds to DSBs, which then recruits ATM and TIP60. **b**) Activated ATM phosphorates H2AX, leading to the formation of γH2AX that provides binding sites for MDC1. **c**) Next, two ubiquitin ligases RNF8 and RNF168 are recruited to catalyse polyubiquitination of γH2AX. This ubiquitination event is tightly controlled by various positive and negative regulators. **d**) Subsequently, BRCA1 (in the form of BRCA1-A complex) and 53BP1 are recruited; these two proteins play important roles in the balance between HR and NHEJ, wherein a variety of regulatory mechanisms are involved.

HR-mediated repair begins with the recognition and binding of DSB ends by the MRN (MRE11–RAD50–NBS1) complex (41,42) (Figure 1a). Subsequently, MRN recruits a complex of ATM and the histone acetyltransferase TIP60 [TIP60/NuA4 complex mentioned above, which binds to histone H3 methylated at Lys-9 (H3K9me3)], to the sites of damage (43,44). Both its recruitment to DSBs and phosphorylation of TIP60 by c-Abl kinase (20) are required to trigger the acetyltransferase activity of TIP60, leading to the activation of ATM by acetylation-induced auto-phosphorylation (44). The activated ATM then phosphorylates a multitude of substrates in response to DNA damage, particularly H2AX (termed γH2AX when phosphorylated), which serves as an anchoring platform for the accumulation of subsequent HR factors (Figure 1b), and is considered as an early marker of DSB signalling [reviewed in (45,46)]. The recruitment of HR factors at sites of damage is regulated by various post-translational modifications which have been subject of comprehensive reviews (47,48), only some of the relevant post-translational modifications will be highlighted here.

The adaptor protein MDC1 localizes to DSB sites by direct binding to γH2AX [reviewed in (9,45)]. MDC1 also harbours a binding site for NBS1 component of MRN complex, promoting additional ATM recruitment and kinase activation (49,50). The ability of MDC1 to bind γH2AX and NBS1 simultaneously enables positive feed-forward phosphorylation of H2AX by ATM and generates a megabase-sized γH2AX region surrounding DSBs [reviewed in (9,46)].

After its recruitment, MDC1 is phosphorylated by ATM. MDC1 serves an important role as a scaffold for the downstream recruitment of the ubiquitin (Ub) E3 ligases RNF8 and RNF168, which work in tandem to ubiquitylate histone H2A and possibly other factors to create docking sites for Ub-binding proteins [see reviews (51,52,53)]. Among these are 53BP1 (p53-binding protein 1) and BRCA1 (breast cancer type 1 susceptibility protein), both of which are tumour suppressors and play a critical role in the pathway choice between HR and NHEJ (discussed in more detail below). The mechanisms for signal amplification exist due to crosstalk within one pathway and also across different pathways. RNF168 itself has ubiquitin-binding domain and E3 ligase activity, which together provide RNF168 the capability to amplify its own catalytic product. RNF8 but not RNF168 also promotes extensive decondensation of higher-order chromatin structure by recruiting the NuRD component CHD4 (31), which in turn promotes the recruitment and activation of RNF8, RNF168 and subsequent assembly of downstream repair factors [reviewed in (54)]. As discussed in the previous section, PARylation is also required to recruit NuRD to assist chromatin ubiquitination at sites of breaks.

Multiple regulators tightly control RNF8/RNF168-mediated ubiquitination in HR. At present, four DUB enzymes (USP3, USP16, BRCC36 and OTUB1) and two HECT E3 ligases (TRIP12 and UBR5) have been shown to target RNF168 for proteasome-mediated degradation, potentially constraining the DSB repair machinery around the break site, and terminating the signal after repair has completed [reviewed in (51)]. Interestingly, unlike TRIP12 and UBR5, another HECT E3-ligase, HERC2, promotes RNF8/RNF168-based ubiquitination (55). In addition, another E3 ligase, RNF169, has an unexpected negative role in regulating RNF8/RNF168-induced ubiquitin signalling by directly binding to ubiquitin-modified chromatin, leading to impaired recruitment of 53BP1 and BRCA1 [reviewed in (51)]. Moreover, SUMOylation of HERC 2 and RNF8 is also involved in the regulation of RNF8/RNF168-induced ubiquitination (56).

Following RNF8/RNF168-catalysed ubiquitination of DSB-flanking chromatin, BRCA1 and 53BP1, two seemingly antagonistic factors, localize to the DSBs at approximately the same time (Figure 1d), providing an important layer of discrimination for DSB repair pathway choice. BRCA1 is required for functional HR, while 53BP1 promotes NHEJ by preventing DSB-end resection that is essential for HR. Interestingly, loss of 53BP1 can largely relieve the requirement of BRCA1 for HR, suggesting that a major role of BRCA1 in HR is to overcome a barrier to resection posed by 53BP1 (57,58). This finding may have clinical implications, as a recent study showed that loss of BRCA1 often activates 53BP1 degradation in cancer cells (59). Below we summarize current knowledge on how these two proteins are recruited, their role in determining pathway choice, and the regulation mechanisms that are involved.

BRCA1 participates in multiple stages of HR by forming at least three mutually exclusive complexes: the BRCA1-A, BRCC and BRCA1-C complexes by binding to different adaptors (Abraxas, BACH1 and CtIP, respectively) [reviewed in (60,61)]. Following RNF8/RNF168-mediated ubiquitination of H2A and H2AX, BRCA1 is recruited to DSBs in the form of the BRCA1-A complex (61,62). The accumulation of this complex to DSBs takes place through the binding of the Abraxas-RAP80 subcomplex to K63 polyubiquitin chains catalysed by RNF8 and RNF168 (63–65). SUMOylation of BRCA1 mediated by PIAS1 and PIAS4 is thought to promote the recruitment of the BRCA1-A complex, and stimulates the ubiquitin ligase activity of BRCA1 (66,67).

53BP1 does not contain any known ubiquitin-binding motif and its accumulation at DSBs relies on binding to methylated histone H4 (68) and ubiquitinated histone H2A, the latter being a product of the RNF168 ubiquitin ligase activity (69). In addition, post-translational modifications of p53BP1 itself, including PIAS1/PIAS4-mediated SUMOylation, can promote the recruitment of 53BP1 at sites of DSBs (67)

The regulation of DSB-repair pathway choice comes from the actions of 53BP1 and RIF1. Several recent studies have elegantly demonstrated that RIF1 is a downstream effector of 53BP1 in this process. In G1, RIF1 is recruited to DSB sites *via* ATM-dependent 53BP1 phosphorylation, and the 53BP1-RIF1 pathway inhibits the recruitment of BRCA1 to damage sites *via* an unknown mechanism to ensure repair through NHEJ. However, in S and G2 phases, CDK-and ATM-dependent phosphorylations of CtIP (CtBP-interacting protein) support the formation of the CtIP–MRN–BRCA1 (BRCA1-C) complex which displaces RIF1 at break sites to promote DNA resection (70–73). However, unlike 53BP1, the loss of RIF1 only partially rescues HR defect in *BRCA1*-deficient cells, suggesting that additional RIF1-independent activities of 53BP1 might exist. Accordingly, a recent study (74) showed that PTIP is required for 53BP1-mediated inhibition of HR in *BRCA1*-deficient cells, but is dispensable for NHEJ during CSR. Thus RIF1 and PTIP separate 53BP1 functions in productive and pathological DSB repair (74).

Compared to the mechanisms that regulate the assembly of early HR repair factors at DSB sites, those that regulate their disassembly remain largely unknown. The mechanism best-documented so far is the removal of MDC1 from DSB sites through PIAS4-mediated SUMOylation and consequent ubiquitination by the SUMO-targeted E3 ubiquitin ligase RNF4 (75–77), which leads to MDC1 degradation. MDC1 removal is important to remove 53BP1 from the damage sites, and is required for the recruitment of downstream HR proteins such as CtIP, RPA (replication protein A) and RAD51 (DNA repair protein RAD51 homolog 1) (75–77). In addition, TIP60-dependent histone H4 acetylation, which reduces the binding of 53BP1 to methylated histone H4, leads to reduced 53BP1 association with DSB-flanking chromatin (78).

#### DSB end resection

**Figure 2.**
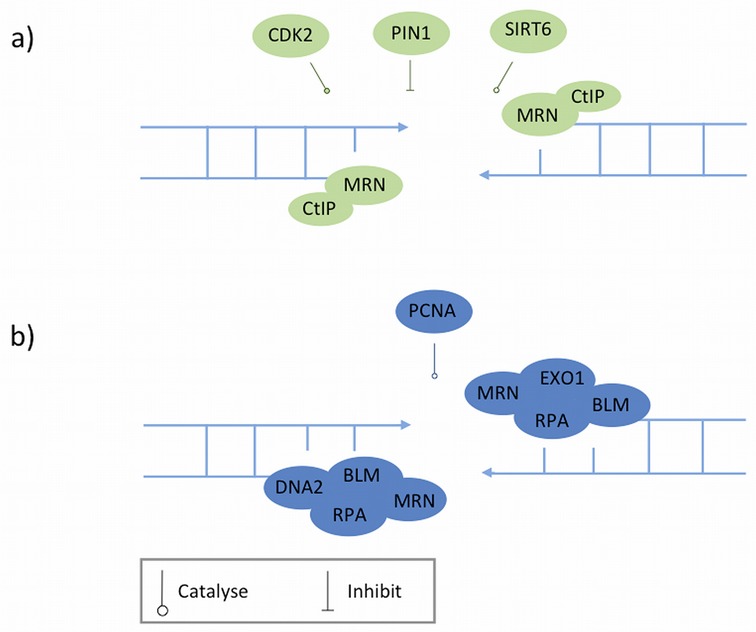
A two-step model for the double-strand break (DSB) end resection. Proteins represented in different colours are recruited at different stages. **a**) The first step, 'initial resection', is carried out by the endonuclease activity of the MRN (MRE11–AD50–NBS1) complex and promoted by CtIP. Multiple regulatory mechanisms, especially the cell cycle-dependent regulation are involved. **b**) The second step, long-range resection, is performed by EXO1 or BLM in concert with DNA2. It remains unclear whether EXO1 and BLM work in parallel or interact.

The sequential recruitment of early-stage HR factors, as outlined above, is required for and followed by DSB end resection—an evolutionarily conserved process that involves 5′-to-3′ nucleolytic degradation of DSB ends to generate 3′ overhangs [a long stretch of single-stranded DNA (ssDNA) at DSB ends; also known as the 3′ tail] (Figure 2). This 3′ overhang is a key determinant of DSB repair pathway choice, which commits cells to HR and is also required for activation of the ATR-mediated checkpoint response (79).

A two-step model has been suggested to describe DSB end resection in mammals (80). The first step, initiation of resection, involves a limited resection that removes ∼50–100 nucleotides from the DSB ends, creating a short 3′ overhang that is further processed in the second step of resection generating a long 3′ overhang essential for the strand invasion step in HR [discussed below; for a recent review see (81)].

The major resection machinery involved in first step is the MRN complex, which has an essential role in damage detection and ATM signalling, in conjunction with CtIP (82,83) (Figure 2a). The initial resection *per se* is carried out by the endonuclease activity of the MRN complex followed by its exonuclease activity (84). CtIP promotes initial resection by interacting with MRN (79) and stimulating its endonuclease activity (83). The activity of CtIP in HR is regulated by multiple mechanisms, among which cell cycle-dependent regulation is of greatest importance because DSB resection must be restricted to the S and G2 phases where sister chromatids are present to serve as templates for HR. In the G1 phase, the level of CtIP protein is suppressed by proteasome-mediated degradation, which is subsequently alleviated as cells enter S phase (85). During S and G2 phases, CtIP is phosphorylated by cyclin-dependent kinases (CDKs) on multiple sites that promote resection in distinct ways. Among them, serine 327 is required for the CtIP-BRCA1 interaction and the formation of the BRCA1-C complex (82,86), and threonine 847 for the localization of CtIP to DSBs and for end resection (87). These CDK-mediated phosphorylation signals directly link the DNA resection capacity with cell cycle control, thereby ensuring that the operation of HR is restricted to the S and G2 phases.

A phosphorylation-specific prolyl-isomerase, PIN1 (peptidyl-prolyl *cis*-trans isomerase NIMA-interacting 1), has recently been shown to counteract CDK-dependent end resection (88). PIN1 controls CtIP levels by promoting its isomerization in a CDK2-dependent manner followed by polyubiquitination (through an as-yet-unknown E3 ubiquitin ligase) and consequent degradation to limit end resection (88).

The second step, long-range resection, is carried out by two alternative pathways involving either the exonuclease function of EXO1 (DNA exonuclease I) alone, or the helicase function of BLM (Bloom syndrome, RecQ helicase-like) in concert with the nuclease function of DNA2 (DNA replication helicase 2) (89–91) (Figure 2b). It remains controversial whether BLM and EXO1 pathway work in parallel (90) or interact [(89); reviewed in (81)]. Recently, CDK1/2 has been shown to promote long-range resection by directly phosphorylating EXO1 on four different sites in mammalian cells (92).

Although PCNA (proliferating cell nuclear antigen) has recently been proposed to facilitate long-range resection by promoting the function of EXO1 (93), in general the regulatory mechanisms involved in this step are not well understood. Interestingly, PCNA is also involved in base excision repair (BER) (94), nucleotide excision repair (NER) (95), mismatch repair (96), translesion synthesis (97), the Fanconi anaemia (FA) pathway (98) and the DNA repair synthesis step as well as suppressing inappropriate recombination in HR (99) (discussed below).

#### D loop formation and DNA repair synthesis

**Figure 3.**
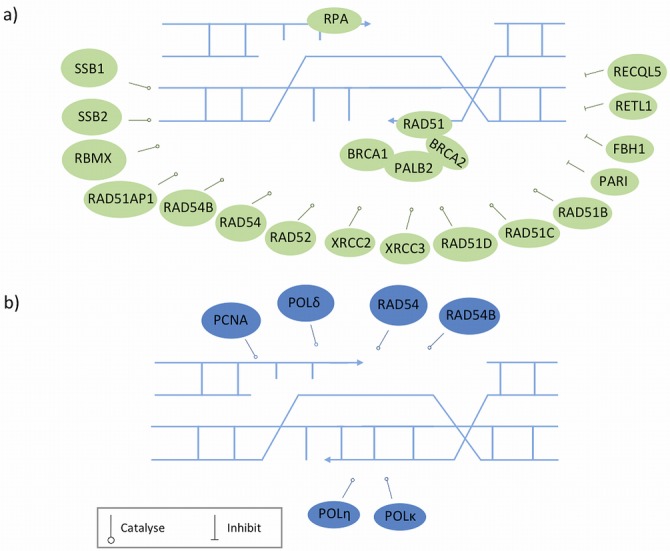
D loop formation and DNA repair synthesis. Proteins represented in different colours are recruited at different stages. **a**) The 3′ ssDNA overhang generated by DSB end resection is coated and stabilized by RPA, which is then displaced by RAD51 with the help of recombination mediators that promote both the formation and stability of RAD51-ssDNA filament. The balancing act of proteins involved in stability and dismantling of RAD51 filaments is depicted here as discussed in the text. Rad51 presynaptic filament performs homology searches with help of other proteins and invades nearby homologous duplex DNA template, resulting in the formation of the D loop structure. **b**) The invading strand is then elongated by copying missing genetic information from the template molecule, which involves the participation of several redundant DNA polymerases.

The 3′ overhang formed by end resection is coated and stabilized by RPA, which prevents ssDNA from forming secondary structure. RPA is then displaced by the evolutionarily conserved recombinase RAD51. The loading of RAD51 onto ssDNA is a critical step in HR, as it generates a nucleoprotein filament that searches for and invades a nearby homologous duplex DNA template (usually a sister chromatid). As a consequence of this invasion, the second strand of the sister chromatid is displaced and a transient structure known as the D (displacement) loop is formed [reviewed in (100,101)] (Figure 3a).

The loading of RAD51 onto ssDNA is promoted and controlled by multiple mechanisms [for a recent review, see (101)]. BRCA2 is the major recombinase accessory factor (also known as recombination mediator) that facilitates the loading of RAD51 onto ssDNA by overcoming the inhibitory effect of RPA (102). PALB2 is a partner and localizer of BRCA2, and serves as a molecular adaptor between BRCA1 and BRCA2 to form the BRCC complex (103,104). In this complex, BRCA1 is thought to fine-tune HR in part through its modulatory role in the PALB2-dependent loading of the BRCA2–RAD51 repair machinery at DNA breaks (103,104). In addition, DSS1 (deleted in split hand/split foot 1), which forms a complex with BRCA2, is required for the stability of BRCA2 and facilitates the role of BRCA2 in RAD51–ssDNA filament formation (105,106).

Recently, the SWI5–MEI5 complex was identified as an evolutionarily conserved mediator of RAD51 (107). This complex contributes to maintenance of the RAD51 nucleofilament in its active ATP-bound form by promoting the release of ADP from this structure (108).

The loading of RAD51 onto ssDNA and subsequent formation of the D loop also depends on the concerted action of other proteins, which include the five RAD51 paralogs (RAD51B, RAD51C, RAD51D, XRCC2 and XRCC3) [reviewed in (109)], RAD52 [RAD52 homolog (S. cerevisiae)] (110), RAD54 [RAD54 homolog (*S*. *cerevisiae*)] and its paralog RAD54B [RAD54 homolog B (*S*. *cerevisiae*)] [reviewed in (111)], RAD51AP1 (RAD51 associated protein 1) (112,113), and the two ssDNA-binding proteins SSB1 (single-strand DNA-binding protein 1) and SSB2 (single-strand DNA-binding protein 2) (114,115).

Although HR has a key role in maintaining genome stability, its aberrant activity can cause genomic instability potentially even leading to cancer. Several anti-recombinases suppress uncontrolled HR activity. These include PARI (PCNA-associated recombination inhibitor), RTEL1 (regulator of telomere elongation helicase 1), RECQL5 (RecQ protein-like 5) and FBH1 (F-box DNA helicase 1). PARI can disrupt toxic RAD51-ssDNA filaments in a PCNA-dependent manner (116), and overexpressed PARI has been implicated in the development of pancreatic cancer (117). RECQL5 regulates HR by targeting undesirable RAD51-ssDNA filament, and is important for tumour suppression in mice (118). FBH1 also functions by targeting RAD51-ssDNA filaments, and its activity in HR is tightly controlled by PCNA (119,120). RTEL1 can suppress inappropriate HR by promoting D loop disassembly (121).

Following D loop formation, the 3′ end of the invading strand serves as a primer for elongation of this strand *via* copying missing genetic information from the template molecule (100) (Figure 3b). For elongation to start, RAD51 at the 3′ end of the invading strand must be removed by RAD54 and RAD54B to reveal the 3′ hydroxyl group for priming (111). The DNA replication machinery involved in this elongation has not been well characterized. Recently Sebesta *et al.* showed that replicative DNA polymerase δ and two TLS polymerases (ν and κ) play redundant roles in strand extension, and PCNA may act as a regulatory point for the recruitment of various polymerases and recombination outcomes (99).

**Figure 4.**
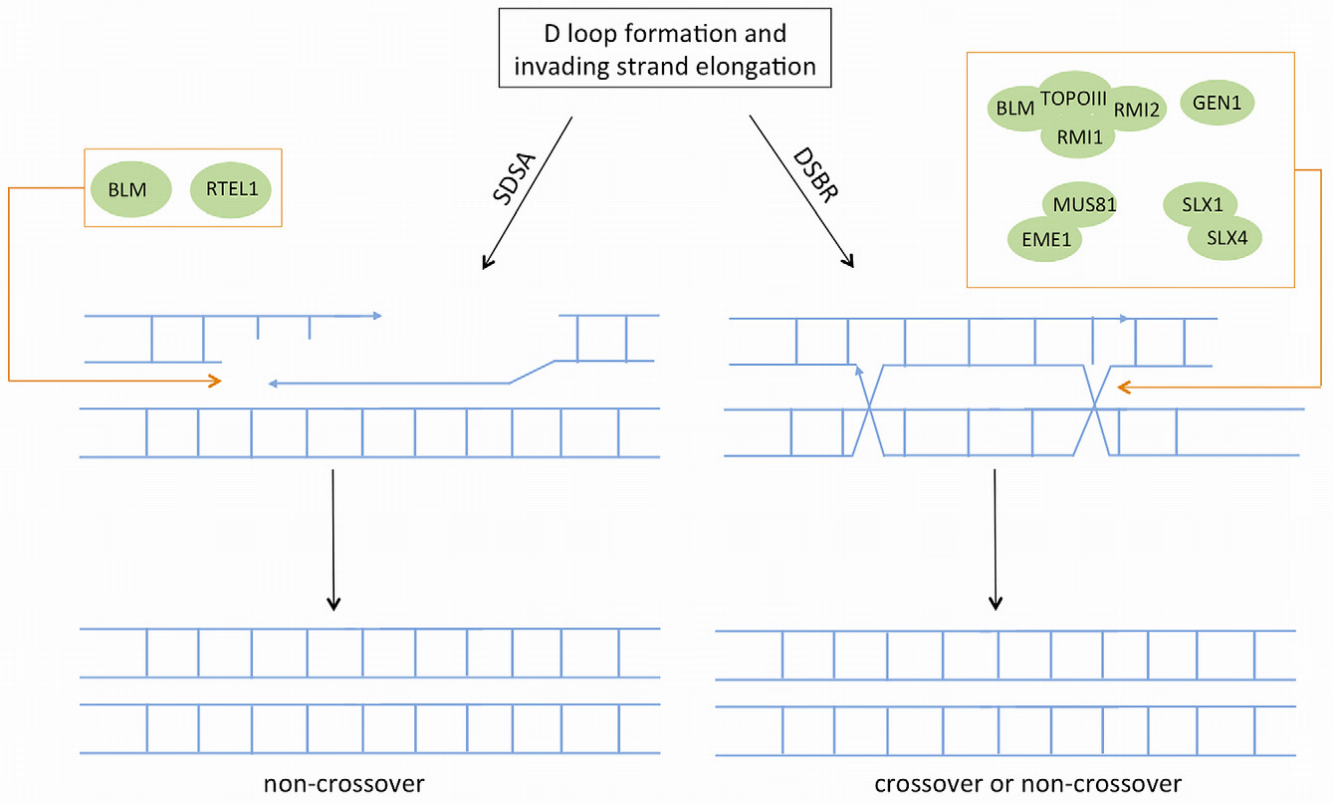
The SDSA (synthesis-dependent strand annealing) and DSB repair sub-pathways. D loop formation and DNA repair synthesis can follow two different routes namely SDSA and DSBR to complete homologous recombination. In SDSA invading strand is displaced from D-loop and annealed with complementary strand associated with second end of the DSB. SDSA is preferred over DSBR during mitosis, and mainly results in a non-crossover product. In the DSBR pathway, the other end of the DSB is captured and double Holliday Junctions (dHJs) intermediate is formed which is then resolved to produce cross-over (mainly during meiosis) or non-crossover products.

HR can take two alternative routes beyond this point (Figure 4). Most frequently, in mitotic cells, elongation of the invading strand continues over only a limited distance, which is then released and anneals with the complementary ssDNA strand associated with the other DSB end. DSB repair is subsequently completed by gap-filling DNA synthesis and ligation. This sub-pathway is referred to as the SDSA (synthesis-dependent strand annealing) pathway (100). RTEL1 is the major enzyme that promotes the disassembly of the D loop structure, resulting in non-crossover products (*i.e.* there is no exchange of genetic information between the original DNA molecule and the template DNA molecule) (121). The D loop can also be processed by BLM to generate a non-crossover product (122), or by the MUS81-EME1 complex to generate a crossover product (123,124)

Alternatively, in the DSB repair sub-pathway typical of meiosis, the second end of the DSB is captured to form an intermediate that harbours two Holliday junctions (HJs) [see reviews (7,100)]. Processing/resolution of the HJs is promoted by various redundant enzymes including the BLM–TOPOIII-RMI1–RMI2 complex (125) and the endonucleases GEN1 (GEN endonuclease 1) (126), the MUS81–EME1 complex (123,124) and the SLX1–SLX4 complex (127) (SLX4 is also known as FANCP in FA). In mitotic cells, the BLM–TOPOIII–RMI1–RMI2 complex is the major machinery responsible for dissolution of HJs to generate a non-crossover product (128,129). Alternatively, HJs can be resolved by endonucleases that simply cleave HJs to generate crossover or non-crossover products. A recent study suggests two redundant pathways of HJ resolution in human cells: one pathway involves GEN1 and the other involves the coordinated action of SLX1–SLX4 and MUS81–EME1 (130). However, another recent study indicated that GEN1 alone cannot replace the resolvase activity provided by SLX1–SLX4 and MUS81–EME1 (131).

#### The SSA sub-pathway

**Figure 5.**
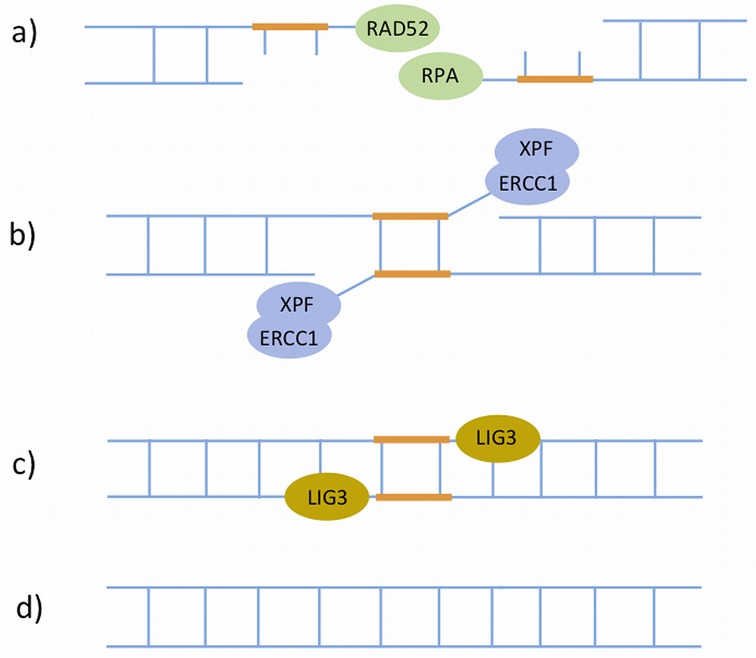
The single-strand annealing (SSA) sub-pathway of homologous recombination. This is a Rad51-independent sub-pathway of HR, which operates when there are regions of homology or direct repeats at both sides of the DSB. **a**) SSA is initiated by RAD52 that binds the 3′ ssDNA ends generated by DSB end resection. RAD52 then functions in concert with RPA to facilitate strand annealing between the two direct repeats. **b**) Next, the XPF–ERCC1 heterodimers remove the non-homologous 3′ single-stranded flaps between the two repeats. **c**) The two DSB ends are re-joined by DNA ligase III. **d**) The sequence continuity is restored.

In addition to canonical HR, an alternative form of HR called SSA has been described (Figure 5). SSA is efficient in repairing DSBs between two direct repeat sequences flanking the ends of the DSB, and results in deletion of sequence between the two repeats. This pathway can be important for both DNA repair and mutagenesis, given that almost half of the human genome consists of repeated sequences (7,100). The activity of SSA has been observed to increase in case of *BRCA2* or *RAD51*-deficiency (132).

SSA is initiated by RAD52 that binds the 3′ ssDNA ends generated by DSB end resection (the same process as described previously), and functions in concert with RPA to facilitate strand annealing between the two direct repeats (133). This is followed by the removal of non-homologous 3′ single-stranded flaps between the two repeats (Figure 5), which is catalysed by a XPF–ERCC1 heterodimer that harbours 5′-3′ structure-specific endonuclease activity (134). In addition to SSA, XPF–ERCC1 also plays an important role in other DNA repair pathways including NER, FA and Alternative NHEJ (discussed below). The final step of SSA is the ligation of the two DSB ends, which is carried out by LIG3 (DNA ligase III) (135).

### Non-homologous end joining (NHEJ)

NHEJ repairs the majority of DSBs throughout the cell cycle in human cells, although it remains unclear why such a low-fidelity pathway has evolved to dominate DSB repair. It is now generally accepted that there exist two forms of NHEJ: canonical and alternative.

#### Canonical NHEJ (C-NHEJ)

**Figure 6.**
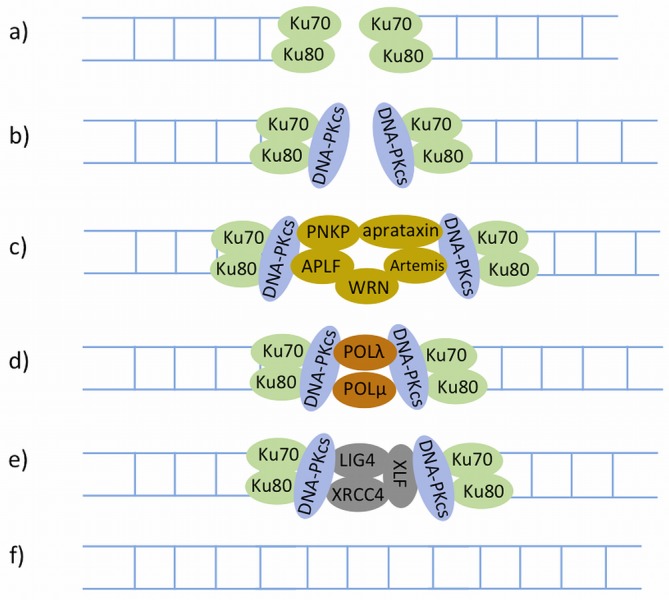
The canonical NHEJ (C-NHEJ). Proteins represented in different colours are recruited at different stages. **a**) The C-NHEJ pathway is initiated by the Ku70–Ku80 heterodimer. **b**) The Ku70–Ku80 dimer then recruits the DNA-PKcs kinase. **c**) In many instances ends of the breaks are not amenable to direct ligation and must be resected or filled in prior to ligation by end processing. **d**) The synthesis step is catalysed by DNA polymerase μ and λ. **e)** The gap after DNA repair synthesis is ligated by the XRCC4–LIG4–XLF complex. **f**) The sequence continuity is restored.

The most common amongst the two pathways, C-NHEJ (136–140) (Figure 6) commences with the rapid recognition and binding of the Ku heterodimer (consisting of Ku70 and Ku80) to DSBs (139,141), which protects and stabilizes the DNA ends, and serves as a scaffold onto which other NHEJ factors can dock (139).

Once Ku is bound to DSB ends, it directly recruits the DNA-PKcs kinase (DNA-dependent protein kinase catalytic subunit) to the damage sites (142), leading to activation of the kinase activity of DNA-PKcs (138,139,143). It has been shown *in vitro* that DNA-PKcs can phosphorylate a large number of NHEJ proteins, but *in vivo* only Artemis (144) and DNA-PKcs itself (auto-phosphorylation) (142) have been demonstrated so far as true substrates of DNA-PKcs phosphorylation [reviewed in (138)].

Ku also directly recruits a complex composed of XRCC4 (X-ray cross complementing protein 4), LIG4 (DNA ligase IV) and XLF (XRCC4-like factor) (145,146) to ligate DNA ends. This recruitment is independent of the presence of DNA-PKcs (146). XRCC4 has no known enzymatic activity in NHEJ, and may serve as a second scaffold for the recruitment of other DSB-processing enzymes in this pathway. In addition, XRCC4 and XLF can form a filament that may play a role in bridging DSB ends (138,147).

In many instances the ends of a DSB are not amenable to direct ligation. For instance, the 5′ hydroxyls or 3′ phosphate termini of a DSB may be covalently modified or the ends may harbour 5′ or 3′ overhangs that must be resected or filled in prior to ligation. Important end-processing factors include PNKP (polynucleotide kinase-phosphatase), Aprataxin, Ku, APLF (aprataxin-and-PNK-like factor), Artemis and WRN (Werner syndrome) [reviewed in (138)]. Specifically, PNKP (148), Aprataxin (149) and Ku (150) remove blocking end groups such as non-ligatable 5′ hydroxyls or 3′ phosphates, as well as abasic sites near DSBs. APLF (151), Artemis (152,153) and WRN (154) have roles in resecting DNA ends [reviewed in (138)]. APLF also facilitates the recruitment and/or retention of the XRCC4–LIG4-XLF complex at DSBs (155).

Following the removal of blocking end groups and DNA end resection, the resulting DNA gaps are filled by the action of DNA polymerases μ and λ, and are then ligated by LIG4 in conjunction with XRCC4 and XLF to finalize this pathway (156).

#### Alternative NHEJ (A-NHEJ)

Like C-NHEJ, A-NHEJ has no inherent mechanism to ensure the restoration of the original DNA sequence in the vicinity of DSBs. Initial evidence for the existence of an alternative form of C-NHEJ, termed A-NHEJ, emerged when C-NHEJ is disabled [see reviews (136,137,140,157)], but a recent study has shown that substantial activity of this pathway can be observed when HR and C-NHEJ are still functional (158). A-NHEJ often benefits from microhomology in the proximity of DSBs; it has been frequently referred to as microhomology-mediated end-joining (MMEJ), but not all A-NHEJ requires microhomology for function (159).

**Figure 7.**
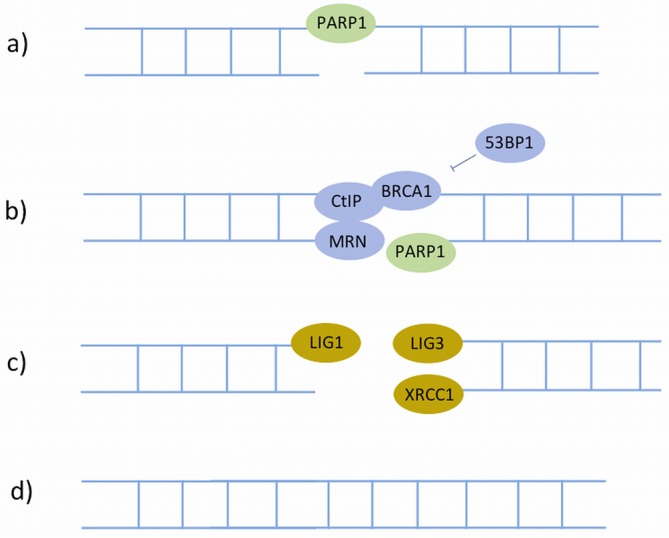
The Alternative NHEJ (A-NHEJ). Proteins represented in different colours are recruited at different stages. In A-NHEJ, **a**) the broken ends are detected and bound by PARP1. **b**) This is followed by end-processing by MRN, CtIP and BRCA1, which is prohibited by 53BP1. **c**) The ligation step can be performed by either LIG3 in concert with XRCC1, or LIG1. **d**) The sequence continuity is restored.

A-NHEJ (Figure 7) is initiated by PARP1, which competes with Ku for binding to DSB ends (160,161). Following this binding, MRN, CtIP and BRCA1 are recruited to the damage sites for end resection (162–166), but this process can be blocked by 53BP1 to promote C-NHEJ to increase repair accuracy (167,168). The step that finalizes A-NHEJ is ligation. Unlike C-NHEJ, which exclusively utilizes LIG4, ligation in A-NHEJ is carried out by either LIG3 (169,170) in a complex with XRCC1 (171), or LIG1 (DNA ligase I) (170,172).

### DSB-repair proteins in replication fork restart

A major physiological source of DNA damage in all cells and at every cell cycle is DNA replication. Replication forks are vulnerable to stalling or collapse (disassembly) due to obstacles encountered during replication, which can be unrepaired DNA damage or presence of DNA-bound proteins or secondary structures. A stalled fork is capable of resuming replication (replication fork restart), whereas a collapsed fork has become inactivated, possibly converting into DSBs that are repaired by HR. While a complex set of pathways from the core replication as well as fork-restart machinery are involved in the resumption of replication, several members of DSB-repair pathways, in particular HR, are known to be involved in this process to varying extents. The roles of these proteins here are distinct from the conventional HR activated during the S-phase. A detailed description of replication fork restart is beyond the scope of this article, and readers are directed to excellent reviews (23,173,174); here we summarize the roles of DSB-repair proteins in this process.

In case of shorter stalls (2–4 h), most replication forks resume progression, with restart promoted by the proteins BLM, WRN, SMARCAL1, PARP1, XRCC3 and RAD51 (23). However, replication forks stalled for many hours (24 h or more) are collapsed and DSBs are generated by the MUS81–EME1 complex (175), following which replication is resumed by new origin firing. The DSBs so-formed promote RAD51-dependent SDSA repair. In addition, PARP1, MRE11, BLM and WRN promote restart of collapsed forks. This suggests that DSB formation by MUS81, and DSB repair-mediated fork restart might be a mechanism to achieve replication fork progression, especially after prolonged fork stalling.

## IMPLICATIONS OF DNA REPAIR FOR TUMORIGENESIS AND CANCER THERAPY

At its core, cancer is a disease driven by genomic instability, accumulating into aberrations in large regions of the genome. Many of these aberrations are hallmarks of erroneous joining of DSB ends, resulting from disruption of DNA repair machinery. These defects, in turn acquired through certain 'driver' events such as mutations, copy-number changes or chromosomal rearrangements cause inactivation of DNA-repair, tumour-suppressor and pro-apoptotic genes, leading to deficiency, misrepair or defects in the repair of DNA damage. Therefore an in-depth characterization of the DSB-repair mechanisms and associating DSB-repair genes to specific driver events in cancer is crucial to understand cancer mechanisms and develop novel therapeutic strategies.

### The genomic landscape of breast cancer

Germline mutations in DNA repair genes are major contributors to familial breast and ovarian cancer development (Table 1). For example, recent estimates suggest that 55–65% of women who inherit a deleterious *BRCA1* mutation, and around 45% who inherit a deleterious *BRCA2* mutation, will develop breast cancer by the age of 70 (176,177). Patients who carry *BRCA1*/*2* mutations are also at a higher risk of developing contralateral disease (178). Likewise, germline mutations in *ATM* result in the autosomal recessive disorder Ataxia-telangiectasia, a neurodegenerative disorder characterized by hypersensitivity to ionizing radiation and a 100-fold increased risk of developing cancer (179). Heterozygous carriers of certain mutations in *ATM* also have a moderate risk of developing breast cancer (180).

**Table 1.** DDR genes associated with breast cancer development, compiled from TCGA and COSMIC

Gene	Gene name	Function of encoded protein	Chromosome band	Somatic mutation frequency in TCGA (%)	Somatic mutation frequency in COSMIC (%)	Copy-number alterations frequency in TCGA (%)	Target of germline mutations, epigenetic changes or SNPs (GWAS locus^†^)
*TP53*	Tumour protein p53	Tumour suppressor involved in cell cycle arrest, apoptosis, senescence and DNA repair	17p13.1	23.15	29.0	0.60↓	Germline (230,231)
*MLL3*	Myeloid/lymphoid or mixed-lineage leukaemia 3	Part of ASCOM complex regulated by acetylation toinduce expression of p53 targets such as p21 in response to DDR (232,233)	7q36.1	4.61	6.48	0.40↑	
*BRCA2*	Familial breast/ovarian cancer gene 2	HR-mediated DSB repair	13q12.3	2.79	2.81	1.70↑↓	Germline (234) and GWAS locus
*PTEN*	Phosphatase and tensin homolog	Tumour suppressor with role in DNA repair through interactions with Chk1 and P53 pathways and regulation of RAD51 activity	10q23.3	2.30	9.13	1.80↓	Germline (235)
*ATM*	Ataxia-Telangiectasia Mutated	Master controller of cellular responses to DNA damage, regulates various tumour suppressors including P53 and BRCA1	11q22-q23	2.06	6.18	0.70↑↓	Germline (163,236); epigenetic silencing (237,238)
*BRCA1*	Familial breast/ovarian cancer gene 1	Tumour suppressor with key roles in HR-mediated DSB repair	17q21	1.82	2.19	1.10↓	Germline (239); epigenetic silencing (240)
*AKT1*	v-akt murine thymoma viral oncogene homolog 1	Regulates components of apoptotic machinery, also checkpoint pathway through phosphorylation of CHK1 (241)	14q32.32	1.45	1.17	1.00↑	
*RB1*	Retinoblastoma gene	Tumour suppressor, mediates cell cycle arrest	13q14.2	1.21	4.64	1.30↓	Germline (242)
*BRIP1*	BRCA1 interacting protein C-terminal helicase 1	Involved in HR-dependent DNA repair by association with BRCA1	17q22.2	0.97	1.39	7.50↑	Germline (243)—not confirmed
*CDKN1B*	Cyclin-dependent kinase inhibitor 1B	Cell-cycle progression at G1	12p13.1-p12	0.61	0.48	0.70↑	
*CCND3*	Cyclin D3	Regulates cell cycle G1/S transition	6p21.1	0.61	0.42	1.10↑	
*HIST1H2BC*	Histone cluster 1, H2bc	Core histone playing roles in DNA repair, replication and chromosomal stability	6p22.1	0.48	0.42	1.00↑	
*CHEK2*	CHK2 checkpoint homolog (*S. pombe*)	Cell cycle arrest in response to DNA damage. Interacts and phosphorylates BRCA1for activating DNA repair	22q12.1	0.48	2.57	0.50↑	Germline (244)
*EP300*	300 kDa E1A-Binding protein gene	Regulates transcription *via* chromatin remodelling. Regulated by acetylation in response to DDR (233)	22q13.2	0.36	2.98	0	
*BAP1*	BRCA1 associated protein-1 (ubiquitin carboxy-terminal hydrolase)	Binds to BRCA1 and involved in cell cycle growth, response to DNA damage and chromatin dynamics.	3p21.1	0.24	2.97	0.40↓	Germline (245)—not confirmed
*CCND1*	Cyclin D1	Regulates cell cycle during G1/S, also interacts with a network of repair proteins including RAD51 to regulate HR (246)	11q13	0.12	0.59	14.1↑	GWAS locus (247)
*PALB2*	Partner and localizer of BRCA2	Critical role in HR-mediated repair by recruiting RAD51 and BRCA2 to DSB sites.	16p12.2	0	1.14	1.80↑	Germline (248)

Germline mutations or epigenetic changes associated with breast cancer risk have been observed for some of these genes, while a few also fall close to single-nucleotide polymorphisms (SNPs) linked to breast cancer risk, identified from genome-wide association studies (GWAS) (http://www.genome.gov/gwastudies (229)).

*^†^GWAS locus*—if the gene is noted as the nearest gene to a breast cancer associated SNP identified by a GWAS study. However, it should be noted that unless a reference is given there is no evidence that the gene is the target of that association. Copy number alterations are shown as predominant amplification (↑) and homozygous deletion (↓) in TCGA cases.

The initiating events in sporadic cancer are less-clearly understood, but large-scale integrated molecular profiling of cancer genomes is beginning to reveal complex landscapes of point mutations, copy-number alterations and chromosomal rearrangements that contribute to tumorigenesis (3,4,181–186).

#### Point mutations and copy-number alterations

At the time of writing, the latest census on cancer mutations from COSMIC (http://cancer.sanger.ac.uk/cancergenome/projects/census/) (187) shows 19 genes implicated in breast cancer either by germline or somatic mutations, of which 11 are involved in DDR (Table 1). This list will expand as potential driver genes identified from large-scale sequencing initiatives are validated. For example, The Cancer Genome Atlas (TCGA) (182) identified 35 significantly mutated genes in breast cancer from analysis of 507 tumour genomes, including 10 novel genes *TBX3*, *RUNX1*, *CBFB*, *AFF2*, *PIK3R1*, *PTPN22*, *PTPRD*, *NF1*, *SF3B1* and *CCND3*. This cohort included genomes harbouring deleterious germline variants in breast cancer susceptibility genes involved in DDR (*ATM*, *BRCA1*, *BRCA2*, *CHEK2*, *PTEN* and *TP53*) (Table 1). Similar large-scale sequencing efforts (4,182–186) have demonstrated extreme heterogeneity in mutation profiles, with *TP53* and *PIK3CA* being the most frequently mutated genes, occurring in over 30% of breast tumours, and the remaining genes (*e.g. GATA3*, *CDH1*, *MAP3K1*, *MAP2K4*, *MLL3*, *PTEN*, *AKT1*, *CDKN2A* and *NCOR1*) mutated at frequencies of 10% or less.

In addition to point mutations, most solid tumours display widespread changes in chromosome number (aneuploidy), as well as deletions, inversions, translocations and other genetic abnormalities. By integrated analysis of DNA copy-number alterations and gene expression profiles in 2000 breast cancers, Curtis *et al.* (183) identified 45 regions of the genome that act as copy-number drivers of gene expression in breast cancer. These included known (*MYC*, *CCND1*, *MDM2*, *ERBB2*, *CCNE1*) and putative candidate driver genes (*MDM1*, *MDM4*, *CDK3*, *CDK4*, *CAMK1D*, *PI4KB*, *NCOR1*, *PPP2R2A*, *MTAP* and *MAP2K4*).

#### Chromosomal rearrangements

Chromosomal rearrangements, particularly intra- and inter-chromosomal translocations, may fuse two genes to create an oncogene (*e.g.* *BCR*–*ABL* fusion gene in chronic myeloid leukaemia) or, in a small number of cases, inactivate a tumour suppressor gene (*e.g.TEL*–*AML* fusion repressing the tumour suppressor *TEL1*). Catastrophic rearrangements (*chromothripsis*), which affects local chromosomal regions, can also have similar tumorigenic effects (188–190). Chromothripsis is characterized by highly focal shattering of chromosomes into tens to hundreds of segments (188), leading to focal amplifications, deletions or fusions in chromosomal regions (191).

In an analysis of 24 breast tumours, rearrangements were found in known cancer genes including *BRAF*, *PAX3*, *PAX5*, *NSD1*, *PBX1*, *MSI2* and *ETV6*, each of which is a partner in a fusion gene in several other human cancers. Rearrangements were also found in tumour suppressor genes such as *RB*, *ABC* and *FBXW7*, possibly resulting in gene inactivation (3).

The analyses of rearrangements also revealed striking signatures of defective DNA repair by different pathways. For instance, in the same study of 1821 rearrangement junctions (3642 breakpoints) in 24 breast tumours (3), the segments on either side of each rearrangement junction showed overlapping microhomology immediately adjacent to the junction. Approximately 15% of the rearrangements showed non-templated sequence at the junction. Overlapping microhomology and non-templated sequences at rearrangement junctions are often considered to be signatures of the NHEJ-mediated repair process. In particular, in some of the tumour genomes, rearrangements with zero base pairs of microhomology were most frequent, while in others rearrangements with two or more base pairs were common, indicating at least two variants of NHEJ repair to be operative in different breast tumours. *BRCA1*- and *BRCA2*-associated tumours showed few tandem duplications, indicating that the mechanisms responsible for chromosomal rearrangements in these tumours were distinct from those in triple-negative tumours, which exhibited tandem duplications.

On the other hand, the mechanistic origin of chromothripsis is largely unclear. Although large-scale genome analyses have not identified chromothriptic rearrangements in breast tumours (192), analysis of rearranged regions in glioblastomas, bone and lung tumours have identified a catastrophic event in which chromosomes undergo multiple fragmentation and rejoining, mainly by NHEJ (191,193). Sequencing of samples from primary, relapse and metastatic tumours have noted that most of these chromothriptic events were present in the primary and initial tumours and did not necessarily occur in an on-going basis or only during metastasis (188,194).

#### Molecular basis of breast tumours revealed through mutational signatures

Large-scale sequencing studies such as TCGA and those initiated by the International Cancer Genome Consortium (ICGC) have generated an increasingly comprehensive atlas of molecular alterations across a wide range of cancers and allowing a systematic exploration of the genetic basis of cancer. This has led to studies identifying *mutational signatures* across cancers (195,196). For example, 21 mutational signatures have been identified across ∼7000 tumours (195) associating cancers to risk factors such as exposure to specific carcinogens, particularly smoking in lung cancer and UV radiation in melanoma.

Breast tumours are largely characterized by three signatures (1B, 2 and 3) strongly associated with age, APOBEC activity and *BRCA1*/*2* mutations, respectively. These signatures are predominantly characterized by C>G and C>T changes, and 'rainfall plot' clustering of these mutations exhibits heavily mutated stretches of the genome characterized by distinctive C>T transitions at TpCpX trinucleotides, resembling *kataegis* (Greek for shower or thunderstorm) in these plots (197,198).

The correlation of breast cancer mutations with the age of diagnosis (Signature 1B) is consistent with the hypothesis that a substantial proportion of these mutations are acquired over the lifetime of the patient at a relatively constant rate that is similar in different people. Signature 2 is attributed to the overactivity of the APOBEC family of cytidine deaminases, which convert cytidine to uracil, coupled to activity of the base excision repair and DNA replication machinery. Because APOBEC activation constitutes part of the innate immune response to viruses and retrotransposons, it has been hypothesized that collateral damage on the genome might be initiated from a response originally directed at retrotransposing DNA elements or exogenous viruses (199,200). Finally, Signature 3 is associated with inactivating mutations in *BRCA1* and *BRCA2* genes, indicating that abrogation of functional HR- and/or NHEJ-mediated repair contributes considerably to breast cancer development, even in patients not harbouring a germline mutation in either of these two genes.

Likewise, another large-scale study (196) characterized ∼3000 tumours on the basis of ∼500 selected functional events (SFE) encompassing copy-number gains and losses, recurrent mutations and epigenetic silencing of genes. Based on these SFEs, tumours were classified into two classes, *M* primarily with mutations, and *C* primarily with copy-number alterations, revealing a characteristic trend of 'genome hyperbola'—cancers have either a large number of mutations or a large number of copy-number alternations, but rarely both. Breast cancer was included in class *C*, as reflected in amplifications of the *MYC* oncogene, *CCND1* and *PIK3CA*, deletion of *CDK2NA*, and inactivating mutations in *TP53* leading to copy-number instability. A subclass of tumours in *C* showed copy-number alterations in cell cycle regulation and DDR pathways attributable to amplification of the gene encoding the mitotic regulator AURKA kinase and the inactivation of *BRCA1* and *BRCA2* genes.

Analyses of mutational signatures across cancers have led to three fundamental observations so far (182,195,196): tumours originating in the same organ or tissue vary substantially in the number, type and pattern of genomic alterations; similar patterns of genomic alteration are observed in tumours from different tissues of origin; and common mutational signatures in tumours are 'imprints' of common underlying mechanisms (*e.g.* APOBEC activity or DDR deficiency) or factors (*e.g.* age and exposure to carcinogens/DNA damage). These observations suggest that 'signature-driven therapies' designed and tailored to tissue-specific tumour types could be extensible across classes of cancer that share similar mutational signatures.

### DNA repair pathways as targets for cancer therapy

The efficacy of DNA damage-based therapy can be modulated selectively towards cancer cells by targeting DNA-damage induced checkpoint and repair pathways (21,201,202). Drugs and agents that inhibit the activity of DNA repair pathways have been reviewed in detail elsewhere (6,21,203,204); here we focus on an exciting strategy called *synthetic lethality*, which has recently gained attention due to its potential for being both selective for and highly effective against cancer cells.

#### Synthetic lethality-based therapy

Synthetic lethality refers to a type of genetic interaction in which the co-occurrence of two genetic events results in death of the cell or organism (205,206). For example, two genes are synthetic lethal when their simultaneous inactivation results in cell death, but deletion of either individually does not affect cell viability. Two common models have been proposed to explain synthetic lethality between two genes (207):

- the two genes function in parallel pathways, with each contributing to a process essential to viability, or
- the genes encode proteins that form part of an essential complex that is partially functional in the absence of one of the proteins, but its functions are completely disrupted in the absence of both.

#### Leveraging synthetic lethality to selectively target cancer cells

Cancer cells undergo a multi-step selection for acquisition of hallmark phenotypes including evasion of apoptosis, insensitivity to growth-control signals and unlimited replicative potential (208,209). In this scenario, genes of minor importance to the well-being of normal cells may become essential lifelines specifically in cancer cells, providing opportunities for novel therapeutic interventions (209).

**Figure 8.**
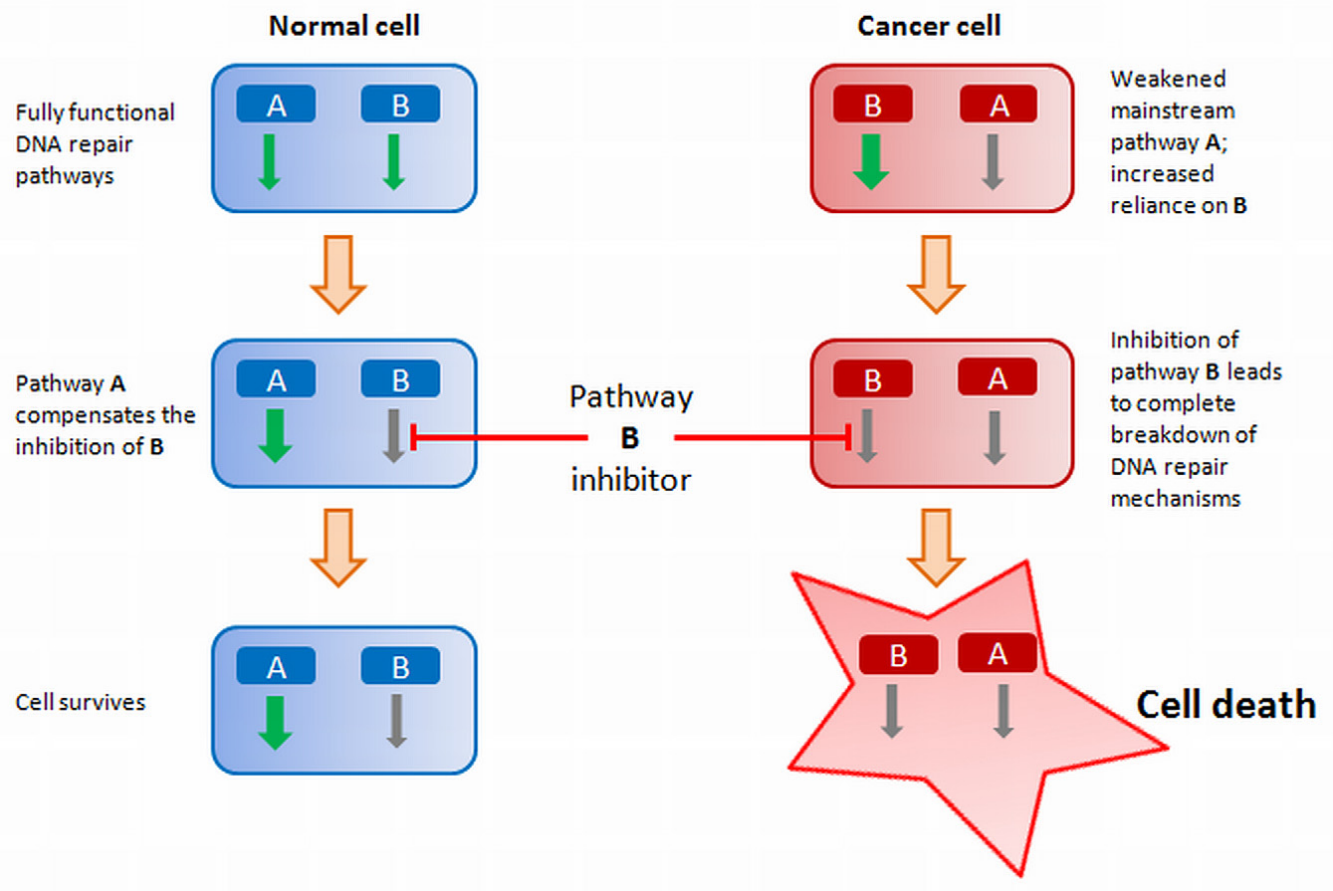
Strategy for synthetic lethality based cancer therapy: targeted inhibition of DNA-damage repair pathways in defined cancer cell populations to selectively kill cancer cells.

The DNA repair machinery is attractive in this context, given that cancer cells are driven by a loss of fidelity in DNA repair and continually accumulate further DNA damage (Figure 8). Selective killing of cancer cells could be made possible either by targeting an otherwise non-essential gene that has turned essential and hence lethal specifically in cancer cells, or alternately by inducing massive amounts of DNA damage (*via* DNA-damaging chemotherapeutic agents or radiation) and subsequently forcing cancer cells into DNA-damage-induced apoptosis. Normal cells remain adequately buffered to repair the induced DNA damage, and will continue to maintain regular function and homeostasis.

#### BRCA1–PARP1 synthetic lethality

A clinically relevant synthetically lethal relationship in the DDR has been documented between mutations in *BRCA1* or *BRCA2* and the inhibition of PARPs (210,211). *BRCA1*- or *BRCA2*-deficient cells are sensitive to siRNA-mediated knockdown or chemical inhibition of PARP, leading to the clinical testing of PARP inhibitors as potential anti-cancer drugs in *BRCA1-* or *BRCA2*-deficient cancers. This suggests a new approach to cancer therapeutics: olaparib (AZD2281), veliparib (ABT-888) and niraparib (MK-4827) are some of the PARP inhibitors that are in advanced clinical trials (212).

Despite the pronounced synthetic lethality observed between BRCA1/2 deficiency and PARP inhibition, the exact mechanism responsible for this observed phenomenon remains somewhat contentious. Nonetheless, the inhibition of PARP itself is not lethal for mammals, and PARP1^−/−^ mice are viable and fertile, even though they manifest accelerated aging and exhibit a higher incidence of tumours compared to wild-type controls (213). The reason PARP1 is non-essential could be due to overlapping functions with other members of the PARP family, in particular PARP2 (214). However, most PARP inhibitors inhibit both PARP1 and PARP2 and the side-effects of this inhibition appear to be mild in both mice and humans (212), suggesting that the pronounced effect of PARP inhibition might be specific to HR-deficient cells.

An early model attributed the pronounced lethality between BRCA1/2 deficiency and PARP inhibition to the involvement of PARP1 in BER. In this model, PARP inhibition leads to persistent accumulation of SSBs, which convert to lethal DSBs during the S-phase; the inability to repair these DSBs in HR-deficient cancer cells result in the selective death of these cells. However, subsequent studies failed to demonstrate an increase in SSBs upon PARP inhibition in BRCA2-deficient cells (215), or reproduce synthetic lethality upon inhibition of XRCC1, an essential component of BER (216), suggesting that this may not be the mechanism of action of this synthetically lethal relationship.

**Figure 9.**
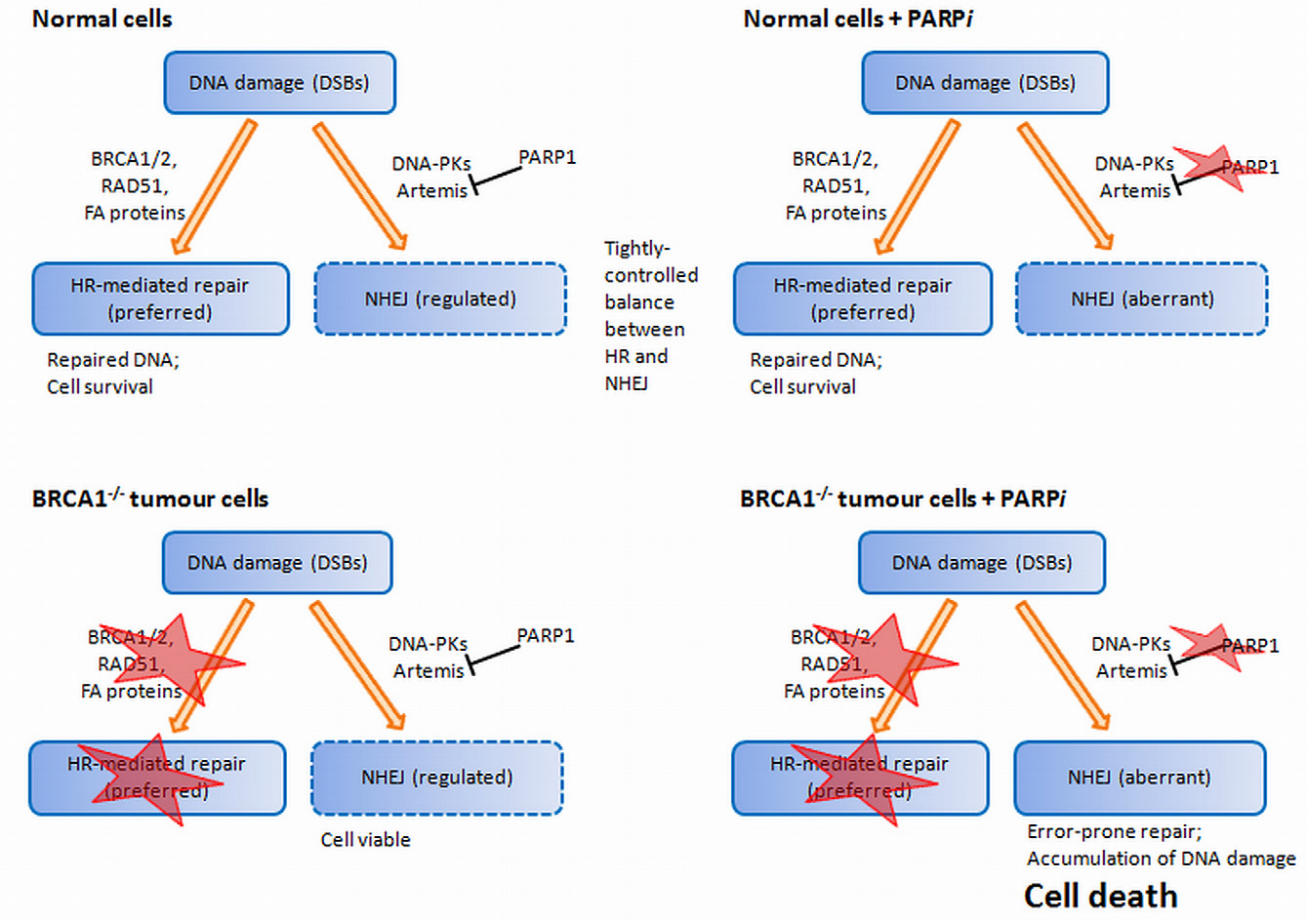
Alternative model (198) centred on the unrestricted error-prone NHEJ as a cause of death in tumour cells. HR-deficient cells were found to be hypersensitive to PARP1 inhibition, but this effect was reversed by disabling C-NHEJ, verified through knockdown of *Ku80* and *Artemis*. This suggests that C-NHEJ contributes to the toxicity of PARP1 inhibitors in HR-deficient cells, and therefore an active C-NHEJ is necessary for PARP inhibitor-based synthetic lethality.

Recent studies suggest that additional roles for PARP in DNA repair may be responsible for this observed synthetic lethality (215–221). The contribution of PARP1 to DSB repair, in particular through its involvement in alternative NHEJ [Alternative NHEJ (A-NHEJ) section], has been suggested for its observed synthetic lethality with HR. A deficiency in HR could further result in lesions that require PARP1-dependent NHEJ for repair. However, PARP inhibition shifts this dependency onto the DNA-PKcs-dependent canonical NHEJ, thereby exposing HR-deficient cells to aberrant repair, resulting in increased genomic instability and apoptosis (216,221) (Figure 9).

In addition to these roles, PARP1 also plays a role at stalled replication forks (DSB-repair proteins in replication fork restart section), and *in vitro* studies in BRCA2-deficient cells suggest that PARP1 protects stalled replication forks from MRE11A-mediated degradation in a manner that is distinct and complementary to the role of BRCA2, resulting in synthetic lethality with BRCA2 at stalled replication forks (219,220).

Further, the chemical action of PARP inhibitors itself can contribute to cell death. Most PARP inhibitors target the catalytic site of the enzyme and thereby block the binding to its substrates, thus preventing PAR-synthesis and causing the enzyme to be 'trapped' on the DNA (222). As a result, PARP inhibition not only restricts its signalling, but the inactivated enzyme forms an obstacle that prevents access for repair proteins to the damaged site or hinders replication (223).

In normal cells, the inhibition of PARP alone is not sufficient to kill these cells as both HR and the canonical NHEJ pathways provide functional repair of DSBs throughout the cell cycle. Cancer cells are prone to excessive oncogene-induced replication stress, often resulting in increased levels of DNA damage (224). An increased PARP activity might be required for protecting stalled replication forks from degradation, fork restart (discussed earlier) or alternative NHEJ-mediated repair of DSBs generated at replication fork, and the increased levels of PARP1 expression seen in cancer cells might be reflective of such PARP activity (210). Therefore, upon PARP inhibition, as demonstrated in BRCA1/BRCA2-deficient cells, HR becomes essential to resolve these lesions (211). Indeed, cells lacking or with inhibited PARP1 display an increase in HR, sister chromatid exchange and micronuclei formation (225,226). It is also possible that various components of HR are in general essential for survival during PARP inhibition, and thus become synthetically lethal to the cell during HR deficiency. In support of this, deficiency in RAD51, MRE11, NBS1, RPA1 and loss of *PALB2* and *RAD51D* has been shown to sensitize cells to PARP inhibition (227).

### DSB repair as a determinant of resistance to cancer therapy

It has long been known that DSB-repair-deficient tumours attain resistance by improving their DSB repair potential (5). In some cases such as breast and ovarian cancer, mutational events in any of the genes (discussed earlier) affect only a subset of the domains of these genes, leaving the remaining domains functional with some residual pathway activity. For example, mammary tumours from *BRCA1^C61G^* mutant mice lacking a functional RING domain respond more poorly to cisplatin than do BRCA1-*null* mammary tumours (228), indicating that a certain basal activity of RING-deficient BRCA1 protein is sufficient to reduce initial drug sensitivity and promote drug resistance (229).

Secondary mutations in these genes can potentially restore their functionality, also contributing to therapy resistance (230,231). For example, *BRCA1-* and *BRCA2*-mutant cells are known to develop acquired resistance to PARP-inhibitor treatment due in part to secondary mutations in these genes that restore the reading frame and produce a functional protein that reverses the HR deficit (230–232). In some of the PARP-inhibitor resistant *BRCA2*-mutant clones the mutation was spliced out, allowing functional BRCA2 proteins to be produced with internal deletions (233,234).

Tumours with intrinsic HR deficiencies may counteract therapeutic sensitivity by rewiring their DNA repair pathways or by altering pathway choices. For example, alterations in the balance between HR and NHEJ may change responses to DSB-inducing agents, as is seen when the loss of 53BP1 resulting from truncating *TP53BP1* mutations confers PARP-inhibitor resistance in *BRCA1*-deficient cells by providing the CtIP protein with unrestricted access to DNA breaks and facilitating DNA end resection (57,58,235). Loss of 53BP1 also restricts NHEJ, which is required for the success of PARP1-inhibitor therapy (216). Likewise, HSP90-mediated stabilization of BRCT domain-mutated BRCA1 protein can confer resistant to PARP inhibitors, reversible by treatment with an HSP90 inhibitor (236). Suppressing NHEJ components including Ku70, Lig4 or DNA-PKcs alters the tight balance between HR and NHEJ, and such a strategy has the potential to be used against FA (237,238).

These observations collectively mean that deeper understanding of the underlying functional relationships, particularly their specific genetic context and alternative rewiring in response to therapy, is critical to counter restoration of DSB repair and hence the development of resistance to therapy. Cancer pathways have been compared to a transport or subway map (209,239): blocking a major commuter line will have repercussions throughout the network as passengers try to find alternative routes to their destinations. Similarly, targeted cancer therapies are thwarted by the emergence of drug resistance, typically through unanticipated rewiring of signalling pathways and the surfacing of alternative functional relationships that are not obvious from the original wiring diagrams (209,240,241).

## CONCLUSION

At its core, cancer is a disease driven by genomic instability, and cancer cells differ genetically from normal cells especially in the ability to repair their DNA. These differences can be exploited to selectively kill cancer cells. However, this requires a deep understanding of the intricacies of DDR pathways, in particular of DSB repair, in order to precisely modulate the pathways and sensitize cancer cells to DSB-inducing drugs.

Here, we have presented an in-depth description of DSB repair mechanisms, focusing on HR and NHEJ, reflecting the latest state of knowledge in the field. We have discussed synthetic lethality as a new strategy to target components of these pathways, with emphasis on the BRCA1–PARP1 relationship that opened up promising avenues for targeted therapies in breast cancer. Finally, we considered cases in which cancer cells become resistant to therapy by improving their DSB-repair potential. These observations suggest that we need better biomarkers to detect patients with HR deficiency eligible for treatment with PARP inhibitors. It is likely that the response to other cancer therapeutics including inhibitors of other repair pathways will also become more predictable, thus allowing more effective, targeted cancer treatments.

## FUNDING

National Health and Medical Research Council (NHMRC) Senior Principal Research Fellowships [1024612 to G.C.T. and 613638 to K.K.K.]; National Breast Cancer Foundation Fellowship, Australia [ECF-10-12 to P.T.S.]; NHMRC Project Grant [1028742 to P.T.S. and M.A.R.].

*Conflict of interest statement.* None declared.
